# *N*,*N*,*N*′,*N*′-Tetra­methyl-*N*′′-[3-(trimethyl­aza­nium­yl)prop­yl]guanidinium bis­(tetra­phenyl­borate) acetone disolvate

**DOI:** 10.1107/S1600536813003024

**Published:** 2013-02-02

**Authors:** Ioannis Tiritiris

**Affiliations:** a Fakultät Chemie/Organische Chemie, Hochschule Aalen, Beethovenstrasse 1, D-73430 Aalen, Germany

## Abstract

In the title solvated salt, C_11_H_28_N_4_^2+^·2C_24_H_20_B^−^·2C_3_H_6_O, the C—N bond lengths in the central CN_3_ unit of the guanidinium ion are 1.3331 (16), 1.3407 (16) and 1.3454 (16) Å, indicating partial double-bond character in each. The central C atom is bonded to the three N atoms in a nearly ideal trigonal–planar geometry [N—C—N angles = 118.96 (11), 120.51 (12) and 120.53 (11)°] and the positive charge is delocalized in the CN_3_ plane. The bonds between the N atoms and the terminal C-methyl groups of the guanidinium moiety all have values close to a typical single bond [1.4601 (16)–1.4649 (16) Å]. In the crystal, the guanidinium ion is connected by N—H⋯O and C—H⋯O hydrogen bonds with the acetone mol­ecules. C—H⋯π inter­actions are present between the guanidinium H atoms and the phenyl rings of both tetra­phenyl­borate ions. The phenyl rings form aromatic pockets, in which the guanidinium ions are embedded.

## Related literature

For the crystal structure of ammonium tetra­phenyl­borate, see: Steiner & Mason (2000 ▶). For the crystal structures of choline tetra­phenyl­borate, triethano­lammonium tetra­phenyl­borate dihydrate and 6-ammonio-*n*-hexa­noic acid tetra­phenyl­borate monohydrate, see: Steiner *et al.* (2001 ▶). For the synthesis of *N*′′-[3-(dimethyl­amino)­prop­yl]-*N*,*N*,*N*′,*N′-*tetra­methyl­guan­id­inium chloride, see: Tiritiris & Kantlehner (2012 ▶). For the crystal structures of alkali metal tetra­phenyl­borates, see: Behrens *et al.* (2012 ▶). For the crystal structure of *N*,*N*,*N*′,*N*′,*N*′′-penta­methyl-*N*′′-[3-(trimethyl­aza­nium­yl)prop­yl]guanidinium bis­(tetra­phenyl­borate), see: Tiritiris (2013 ▶). 
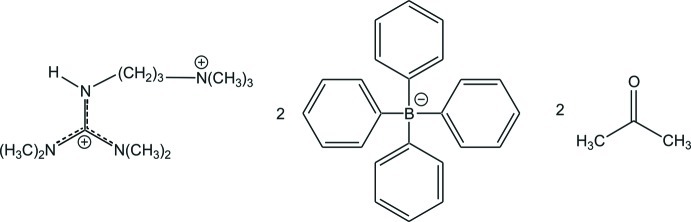


## Experimental

### 

#### Crystal data

C_11_H_28_N_4_^2+^·2C_24_H_20_B^−^·2C_3_H_6_O
*M* *_r_* = 970.95
Monoclinic, 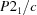

*a* = 19.8630 (5) Å
*b* = 18.3212 (4) Å
*c* = 16.5349 (3) Å
β = 110.763 (1)°
*V* = 5626.5 (2) Å^3^
*Z* = 4
Mo *K*α radiation
μ = 0.07 mm^−1^
*T* = 100 K
0.23 × 0.16 × 0.12 mm

#### Data collection

Bruker–Nonius KappaCCD diffractometer
25784 measured reflections
13908 independent reflections
10017 reflections with *I* > 2σ(*I*)
*R*_int_ = 0.030

#### Refinement

*R*[*F* ^2^ > 2σ(*F* ^2^)] = 0.045
*wR*(*F* ^2^) = 0.113
*S* = 1.02
13908 reflections
674 parameters
H atoms treated by a mixture of independent and constrained refinement
Δρ_max_ = 0.31 e Å^−3^
Δρ_min_ = −0.23 e Å^−3^

### 

Data collection: *COLLECT* (Hooft, 2004 ▶); cell refinement: *SCALEPACK* (Otwinowski & Minor, 1997 ▶); data reduction: *SCALEPACK*; program(s) used to solve structure: *SHELXS97* (Sheldrick, 2008 ▶); program(s) used to refine structure: *SHELXL97* (Sheldrick, 2008 ▶); molecular graphics: *DIAMOND* (Brandenburg & Putz, 2005 ▶); software used to prepare material for publication: *SHELXL97*.

## Supplementary Material

Crystal structure: contains datablock(s) I, global. DOI: 10.1107/S1600536813003024/zl2531sup1.cif

Structure factors: contains datablock(s) I. DOI: 10.1107/S1600536813003024/zl2531Isup2.hkl

Additional supplementary materials:  crystallographic information; 3D view; checkCIF report

## Figures and Tables

**Table 1:** Hydrogen-bond geometry (Å, °) *Cg*1, *Cg*2, *Cg*3, *Cg*4 and *Cg*5 are the centroids of the C42–C47, C48–C53, C18–C23, C36–C41 and C54–C59 rings, respectively.

*D*—H⋯*A*	*D*—H	H⋯*A*	*D*⋯*A*	*D*—H⋯*A*
N3—H3⋯O1^i^	0.87 (2)	2.18 (2)	2.914 (2)	142 (2)
C11—H11*A*⋯O2^ii^	0.98	2.48	3.368 (2)	151
C10—H10*C*⋯*Cg*1	0.98	2.82	3.693 (1)	150
C8—H8*B*⋯*Cg*2	0.99	2.82	3.510 (2)	127
C3—H3*A*⋯*Cg*3^iii^	0.98	2.79	3.359 (1)	118
C2—H2*C*⋯*Cg*4^iv^	0.98	2.61	3.453 (1)	144
C2—H2*A*⋯*Cg*5^iv^	0.98	2.59	3.393 (1)	140

Symmetry codes: (i) 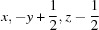; (ii) 
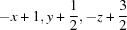
; (iii) 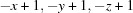; (iv) 
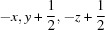
.

**Fig. 1.**
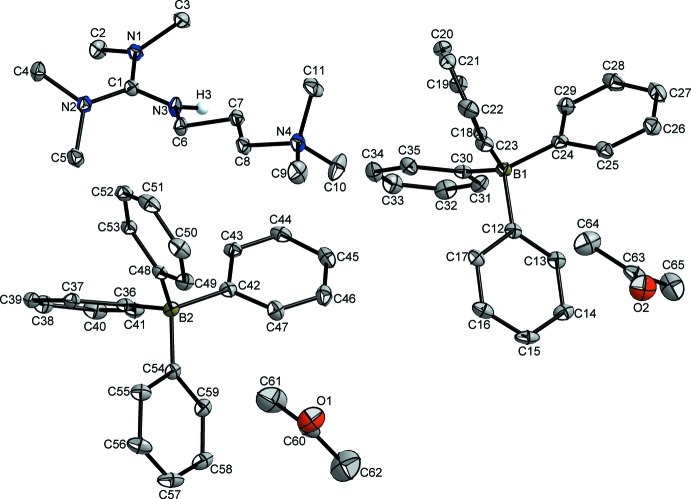
The structure of the title compound with displacement ellipsoids at the 50% probability level. All carbon bonded hydrogen atoms were omitted for the sake of clarity.

**Fig. 2.**
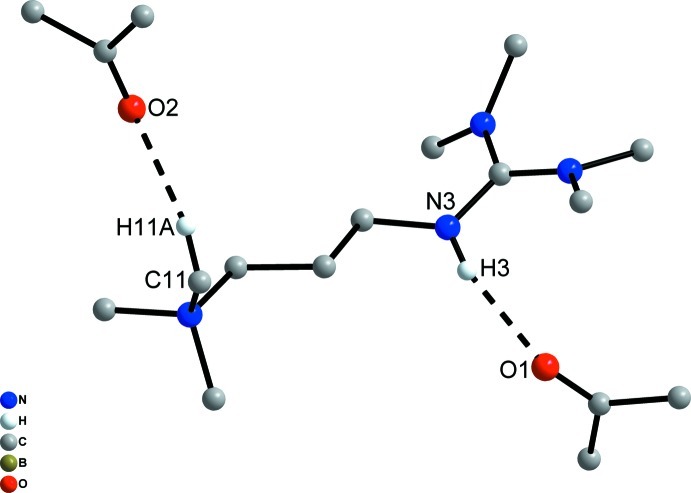
N–H···O and C–H···O hydrogen bonds (dashed lines) between the guanidinium ion and the acetone molecules. The carbon bonded hydrogen atoms (except of H11A) were omitted for the sake of clarity.

**Fig. 3.**
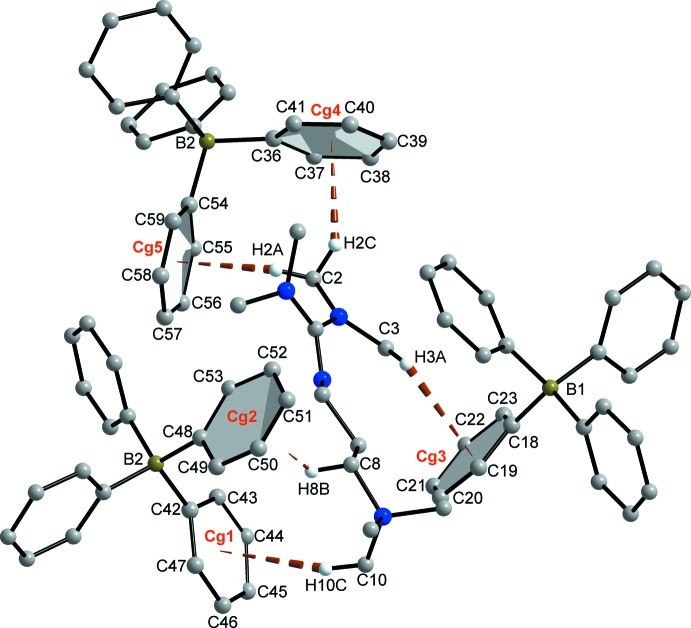
C–H···π interactions (brown dashed lines) between the hydrogen atoms of the guanidinium ion and the phenyl rings (centroids) of the tetraphenylborate ions.

## supplementary crystallographic information

### Comment

*X*–H···Ph hydrogen bonds are found in structural chemistry and structural biology, when donor groups like O–H, N–H or C–H interact with electrons in aromatic π bonds. Tetraphenylborate salts are of great interest, because the (BPh_4_)^-^ ion consists of eight aromatic faces as potential hydrogen bond acceptors. The most prominent model system examined, is ammonium tetraphenylborate [(NH_4_)(BPh_4_)], in which very short aromatic hydrogen bonds (N···*Cg* = 3.023 Å) have been determined in its crystal structure (Steiner & Mason, 2000). Structurally different tetraphenylborate salts with various cations have also been studied, to understand accurately the properties of aromatic hydrogen bonding (Steiner *et al.*, 2001). Guanidinium tetraphenylborates in this context can be similarly interesting systems, but no attention was given to the analysis of the aromatic hydrogen bonding system in those compounds. A peralkylated dicationic guanidinium tetraphenylborate with an additional quarternary ammonium group (Tiritiris, 2013), showed in its crystal structure C–H···π interactions between cationic hydrogen atoms and the aromatic rings of the anions. To examine this type of interactions in similar systems, we synthesized the here presented title compound by *N*-methylation of the corresponding aminoguanidinium chloride (Tiritiris & Kantlehner, 2012) and subsequent anion exchange with NaBPh_4_. According to the structure analysis, the C1–N1 bond of the the CN_3_ unit is 1.3331 (16) Å, C1–N2 = 1.3454 (16) Å and C1–N3 = 1.3407 (16) Å, showing partial double-bond character. The N–C1–N angles are: 120.53 (11)° (N1–C1–N2), 118.96 (11)° (N1–C1–N3) and 120.51 (12)° (N2–C1–N3), which indicates a nearly ideal trigonal-planar surrounding of the carbon centre by the nitrogen atoms. The positive charge is completely delocalized on the CN_3_ plane (Fig. 1). The bonds between the N atoms and the terminal *C*-methyl groups of the guanidinium moiety all have values close to a typical single bond [1.4601 (16)–1.4649 (16) Å]. The C–N bond lengths in the terminal trimethylammonium group are slightly elongated [1.4930 (17)–1.5161 (16) Å]. The bond lengths and angles in the tetraphenylborate ions are in good agreement with the data from the crystal structure analysis of the alkali metal tetraphenylborates (Behrens *et al.*, 2012). In the crystal, the guanidinium ion is connected by N–H···O and C–H···O hydrogen bonds (Fig. 2) with the acetone molecules [*d*(H···O1) = 2.18 (2) Å and d(H···O2) = 2.48 Å] (Tab. 1). In contrast, N–H···Ph interactions with the (BPh_4_)^-^ ions were not detected. Similar to the permethylated compound *N*,*N*,*N*',*N*',*N*''-pentamethyl-*N*''-[3-(trimethylazaniumyl)propyl]guanidinium bis(tetraphenylborate) (Tiritiris, 2013), C–H···π interactions between hydrogen atoms of –N(CH_3_)_2_, –CH_2_ and –N^+^(CH_3_)_3_ groups of the guanidinium ion and phenyl rings (centroids) of both tetraphenylborate ions are present (Fig. 3), ranging from 2.59 to 2.82 Å (Tab. 1). The phenyl rings form aromatic pockets, in which the guanidinium ions are embedded.

### Experimental

The title compound was obtained by reaction of *N*''-[3-(dimethylamino)propyl]-*N*,*N*,*N*',*N*'-tetramethylguanidinium chloride (Tiritiris & Kantlehner, 2012) with one equivalent dimethyl sulfate in acetonitrile at room temperature. After evaporation of the solvent the crude *N*,*N*,*N*',*N*'-tetramethyl-*N*''-[3-(trimethylammonio)propyl]guanidinium chloride methylsulfate (I) was washed with diethylether and dried *in vacuo*. 1.0 g (2.76 mmol) of (I) was dissolved in 20 ml acetonitrile and 1.89 g (5.52 mmol) of sodium tetraphenylborate in 20 ml acetonitrile was added. After stirring for one hour at room temperature, the precipitated sodium chloride and sodium methylsulfate were filtered off. The title compound crystallized from a saturated acetone solution after several days at 273 K, forming colorless single crystals. Yield: 1.97 g (85.4%). ^1^H NMR (500 MHz, CD_3_CN/TMS): δ = 2.19–2.23 (m, 2 H, –CH_2_), 2.98 [s, 12 H, –N(CH_3_)_2_], 3.18 [s, 9 H, –N^+^(CH_3_)_3_], 3.35–3.39 (t, 2 H, –CH_2_), 3.43–3.46 (m, 2 H, –CH_2_), 6.86–6.92 (t, 8 H, –C_6_H_5_), 6.97–7.04 (t, 16 H,–C_6_H_5_), 7.25–7.31 (m, 16 H, –C_6_H_5_). The hydrogen atom of the –NH group was not observed. ^13^C NMR (125 MHz, CD_3_CN/TMS): δ = 26.3 (–CH_2_), 41.5 [–N(CH_3_)_2_], 44.6 (–CH_2_), 55.5–55.8 [–N^+^(CH_3_)_3_], 66.1 (–CH_2_), 122.5 (–C_6_H_5_), 126.2–126.6 (–C_6_H_5_), 136.3 (–C_6_H_5_), 162.8–163.9 (–C_6_H_5_), 164.4 (N_3_C^+^).

### Refinement

The N-bound H atom was located in a difference Fourier map and was refined freely [N—H = 0.87 (2) Å]. The hydrogen atoms of the methyl groups were allowed to rotate with a fixed angle around the C–N bond to best fit the experimental electron density, with *U*(H) set to 1.5 *U*_eq_(C) and d(C—H) = 0.98 Å. The remaining H atoms were placed in calculated positions with d(C—H) = 0.99 Å (H atoms in CH_2_ groups) and (C—H) = 0.95 Å (H atoms in aromatic rings). They were included in the refinement in the riding model approximation, with *U*(H) set to 1.2 *U*_eq_(C).

### Crystal data

**Table:**

C_11_H_28_N_4_^2+^·2C_24_H_20_B^−^·2C_3_H_6_O	*F*(000) = 2096
*M**_r_* = 970.95	*D*_x_ = 1.146 Mg m^−^^3^
Monoclinic, *P*2_1_/*c*	Melting point: 460 K
Hall symbol: -P 2ybc	Mo *K*α radiation, λ = 0.71073 Å
*a* = 19.8630 (5) Å	Cell parameters from 13788 reflections
*b* = 18.3212 (4) Å	θ = 0.4–28.3°
*c* = 16.5349 (3) Å	µ = 0.07 mm^−^^1^
β = 110.763 (1)°	*T* = 100 K
*V* = 5626.5 (2) Å^3^	Block, colorless
*Z* = 4	0.23 × 0.16 × 0.12 mm

### Data collection

**Table:**

Bruker–Nonius KappaCCD diffractometer	10017 reflections with *I* > 2σ(*I*)
Radiation source: sealed tube	*R*_int_ = 0.030
Graphite monochromator	θ_max_ = 28.3°, θ_min_ = 1.1°
φ scans, and ω scans	*h* = −26→25
25784 measured reflections	*k* = −24→24
13908 independent reflections	*l* = −22→22

### Refinement

**Table:**

Refinement on *F*^2^	Secondary atom site location: difference Fourier map
Least-squares matrix: full	Hydrogen site location: difference Fourier map
*R*[*F*^2^ > 2σ(*F*^2^)] = 0.045	H atoms treated by a mixture of independent and constrained refinement
*wR*(*F*^2^) = 0.113	*w* = 1/[σ^2^(*F*_o_^2^) + (0.0447*P*)^2^ + 2.1681*P*] where *P* = (*F*_o_^2^ + 2*F*_c_^2^)/3
*S* = 1.02	(Δ/σ)_max_ < 0.001
13908 reflections	Δρ_max_ = 0.31 e Å^−^^3^
674 parameters	Δρ_min_ = −0.23 e Å^−^^3^
0 restraints	Extinction correction: *SHELXL97* (Sheldrick, 2008), Fc^*^=kFc[1+0.001xFc^2^λ^3^/sin(2θ)]^-1/4^
Primary atom site location: structure-invariant direct methods	Extinction coefficient: 0.0030 (4)

### Special details

**Table:**

Geometry. All e.s.d.'s (except the e.s.d. in the dihedral angle between two l.s. planes) are estimated using the full covariance matrix. The cell e.s.d.'s are taken into account individually in the estimation of e.s.d.'s in distances, angles and torsion angles; correlations between e.s.d.'s in cell parameters are only used when they are defined by crystal symmetry. An approximate (isotropic) treatment of cell e.s.d.'s is used for estimating e.s.d.'s involving l.s. planes.
Refinement. Refinement of *F*^2^ against ALL reflections. The weighted *R*-factor *wR* and goodness of fit *S* are based on *F*^2^, conventional *R*-factors *R* are based on *F*, with *F* set to zero for negative *F*^2^. The threshold expression of *F*^2^ > σ(*F*^2^) is used only for calculating *R*-factors(gt) *etc*. and is not relevant to the choice of reflections for refinement. *R*-factors based on *F*^2^ are statistically about twice as large as those based on *F*, and *R*- factors based on ALL data will be even larger.

### Fractional atomic coordinates and isotropic or equivalent isotropic displacement parameters (Å^2^)

**Table:**

	*x*	*y*	*z*	*U*_iso_*/*U*_eq_	
N1	0.17578 (6)	0.65595 (6)	0.32134 (7)	0.0173 (2)	
N2	0.09127 (6)	0.60442 (6)	0.37204 (7)	0.0180 (2)	
N3	0.20209 (6)	0.54792 (6)	0.39738 (7)	0.0180 (2)	
H3	0.2306 (9)	0.5369 (9)	0.3700 (10)	0.029 (4)*	
N4	0.35625 (6)	0.38934 (6)	0.63758 (7)	0.0184 (2)	
C1	0.15612 (7)	0.60282 (7)	0.36344 (8)	0.0160 (3)	
C2	0.12438 (7)	0.69359 (8)	0.24677 (8)	0.0216 (3)	
H2A	0.0779	0.6683	0.2288	0.032*	
H2B	0.1426	0.6936	0.1989	0.032*	
H2C	0.1183	0.7440	0.2627	0.032*	
C3	0.25152 (7)	0.67090 (8)	0.33660 (9)	0.0218 (3)	
H3A	0.2810	0.6553	0.3952	0.033*	
H3B	0.2581	0.7234	0.3305	0.033*	
H3C	0.2663	0.6441	0.2944	0.033*	
C4	0.05412 (7)	0.67235 (7)	0.37603 (9)	0.0215 (3)	
H4A	0.0879	0.7132	0.3863	0.032*	
H4B	0.0353	0.6696	0.4233	0.032*	
H4C	0.0142	0.6798	0.3212	0.032*	
C5	0.05098 (7)	0.53799 (8)	0.37313 (9)	0.0220 (3)	
H5A	0.0730	0.4966	0.3543	0.033*	
H5B	0.0010	0.5437	0.3339	0.033*	
H5C	0.0519	0.5291	0.4320	0.033*	
C6	0.20631 (7)	0.50449 (7)	0.47324 (8)	0.0172 (3)	
H6A	0.1731	0.4624	0.4553	0.021*	
H6B	0.1915	0.5346	0.5138	0.021*	
C7	0.28311 (7)	0.47731 (7)	0.51828 (8)	0.0173 (3)	
H7A	0.3005	0.4529	0.4760	0.021*	
H7B	0.3154	0.5190	0.5438	0.021*	
C8	0.28381 (7)	0.42392 (7)	0.58888 (8)	0.0189 (3)	
H8A	0.2672	0.4498	0.6309	0.023*	
H8B	0.2487	0.3846	0.5625	0.023*	
C9	0.34691 (8)	0.33815 (9)	0.70388 (9)	0.0296 (3)	
H9A	0.3114	0.3006	0.6748	0.044*	
H9B	0.3301	0.3656	0.7440	0.044*	
H9C	0.3931	0.3149	0.7361	0.044*	
C10	0.38358 (9)	0.34703 (9)	0.57841 (9)	0.0327 (4)	
H10A	0.4277	0.3211	0.6125	0.049*	
H10B	0.3940	0.3805	0.5381	0.049*	
H10C	0.3470	0.3117	0.5458	0.049*	
C11	0.41050 (7)	0.44586 (8)	0.68342 (9)	0.0269 (3)	
H11A	0.3920	0.4751	0.7206	0.040*	
H11B	0.4198	0.4776	0.6409	0.040*	
H11C	0.4554	0.4219	0.7189	0.040*	
B1	0.56358 (7)	0.28698 (8)	0.40853 (9)	0.0154 (3)	
C12	0.54091 (7)	0.20041 (7)	0.40905 (7)	0.0154 (2)	
C13	0.57336 (7)	0.14377 (7)	0.37889 (7)	0.0177 (3)	
H13	0.6108	0.1556	0.3579	0.021*	
C14	0.55300 (7)	0.07087 (7)	0.37838 (8)	0.0211 (3)	
H14	0.5767	0.0343	0.3577	0.025*	
C15	0.49840 (8)	0.05170 (8)	0.40795 (8)	0.0237 (3)	
H15	0.4849	0.0020	0.4087	0.028*	
C16	0.46369 (7)	0.10602 (8)	0.43636 (8)	0.0219 (3)	
H16	0.4255	0.0938	0.4558	0.026*	
C17	0.48465 (7)	0.17872 (7)	0.43652 (8)	0.0187 (3)	
H17	0.4598	0.2151	0.4560	0.022*	
C18	0.56509 (6)	0.33145 (7)	0.49559 (8)	0.0156 (2)	
C19	0.56606 (7)	0.40807 (7)	0.49564 (8)	0.0202 (3)	
H19	0.5604	0.4325	0.4429	0.024*	
C20	0.57492 (7)	0.45017 (8)	0.56874 (9)	0.0231 (3)	
H20	0.5756	0.5019	0.5653	0.028*	
C21	0.58272 (7)	0.41651 (8)	0.64645 (9)	0.0230 (3)	
H21	0.5887	0.4446	0.6968	0.028*	
C22	0.58167 (7)	0.34116 (8)	0.64932 (8)	0.0228 (3)	
H22	0.5868	0.3173	0.7022	0.027*	
C23	0.57314 (7)	0.29968 (7)	0.57563 (8)	0.0188 (3)	
H23	0.5728	0.2480	0.5798	0.023*	
C24	0.64650 (7)	0.29383 (7)	0.40887 (8)	0.0167 (3)	
C25	0.70171 (7)	0.24886 (8)	0.46238 (8)	0.0208 (3)	
H25	0.6900	0.2133	0.4971	0.025*	
C26	0.77284 (7)	0.25417 (8)	0.46677 (9)	0.0255 (3)	
H26	0.8083	0.2224	0.5037	0.031*	
C27	0.79208 (8)	0.30560 (9)	0.41747 (9)	0.0288 (3)	
H27	0.8403	0.3088	0.4194	0.035*	
C28	0.73966 (8)	0.35227 (9)	0.36533 (9)	0.0269 (3)	
H28	0.7521	0.3884	0.3319	0.032*	
C29	0.66869 (7)	0.34620 (8)	0.36189 (8)	0.0211 (3)	
H29	0.6338	0.3791	0.3261	0.025*	
C30	0.50346 (7)	0.32212 (7)	0.32071 (8)	0.0165 (3)	
C31	0.50802 (7)	0.31281 (7)	0.23840 (8)	0.0201 (3)	
H31	0.5489	0.2885	0.2343	0.024*	
C32	0.45556 (8)	0.33743 (8)	0.16284 (8)	0.0241 (3)	
H32	0.4612	0.3299	0.1087	0.029*	
C33	0.39510 (8)	0.37287 (8)	0.16626 (9)	0.0247 (3)	
H33	0.3588	0.3896	0.1148	0.030*	
C34	0.38843 (7)	0.38355 (8)	0.24620 (9)	0.0242 (3)	
H34	0.3475	0.4081	0.2497	0.029*	
C35	0.44161 (7)	0.35837 (7)	0.32122 (8)	0.0198 (3)	
H35	0.4356	0.3661	0.3751	0.024*	
B2	0.08764 (8)	0.27647 (8)	0.42323 (9)	0.0149 (3)	
C36	0.02556 (7)	0.32161 (7)	0.34595 (8)	0.0152 (2)	
C37	−0.03449 (7)	0.35343 (7)	0.35830 (8)	0.0187 (3)	
H37	−0.0376	0.3514	0.4143	0.022*	
C38	−0.08963 (7)	0.38772 (7)	0.29243 (9)	0.0242 (3)	
H38	−0.1290	0.4087	0.3040	0.029*	
C39	−0.08706 (8)	0.39127 (8)	0.20972 (9)	0.0275 (3)	
H39	−0.1240	0.4153	0.1645	0.033*	
C40	−0.02972 (8)	0.35927 (8)	0.19430 (9)	0.0256 (3)	
H40	−0.0276	0.3607	0.1378	0.031*	
C41	0.02485 (7)	0.32498 (7)	0.26076 (8)	0.0195 (3)	
H41	0.0632	0.3029	0.2481	0.023*	
C42	0.16525 (7)	0.27497 (7)	0.40739 (7)	0.0165 (3)	
C43	0.18861 (7)	0.33241 (7)	0.36759 (8)	0.0187 (3)	
H43	0.1589	0.3744	0.3499	0.022*	
C44	0.25350 (7)	0.33058 (8)	0.35282 (8)	0.0219 (3)	
H44	0.2670	0.3708	0.3256	0.026*	
C45	0.29839 (7)	0.27037 (8)	0.37758 (8)	0.0235 (3)	
H45	0.3417	0.2680	0.3656	0.028*	
C46	0.27873 (7)	0.21369 (8)	0.42028 (9)	0.0245 (3)	
H46	0.3095	0.1726	0.4394	0.029*	
C47	0.21397 (7)	0.21661 (8)	0.43538 (8)	0.0215 (3)	
H47	0.2023	0.1775	0.4658	0.026*	
C48	0.10502 (6)	0.31329 (7)	0.51928 (8)	0.0149 (2)	
C49	0.13811 (7)	0.27347 (8)	0.59563 (8)	0.0207 (3)	
H49	0.1463	0.2228	0.5913	0.025*	
C50	0.15947 (7)	0.30496 (8)	0.67742 (8)	0.0244 (3)	
H50	0.1812	0.2757	0.7273	0.029*	
C51	0.14920 (7)	0.37886 (8)	0.68645 (8)	0.0246 (3)	
H51	0.1645	0.4008	0.7421	0.030*	
C52	0.11626 (7)	0.42011 (8)	0.61288 (9)	0.0218 (3)	
H52	0.1086	0.4709	0.6178	0.026*	
C53	0.09428 (7)	0.38738 (7)	0.53158 (8)	0.0166 (3)	
H53	0.0709	0.4167	0.4822	0.020*	
C54	0.05455 (7)	0.19386 (7)	0.41917 (7)	0.0154 (2)	
C55	0.00955 (7)	0.17420 (7)	0.46455 (8)	0.0205 (3)	
H55	0.0006	0.2090	0.5021	0.025*	
C56	−0.02262 (8)	0.10575 (8)	0.45674 (9)	0.0261 (3)	
H56	−0.0531	0.0949	0.4883	0.031*	
C57	−0.01037 (8)	0.05343 (8)	0.40317 (9)	0.0290 (3)	
H57	−0.0317	0.0065	0.3983	0.035*	
C58	0.03344 (8)	0.07060 (8)	0.35682 (9)	0.0257 (3)	
H58	0.0423	0.0353	0.3197	0.031*	
C59	0.06449 (7)	0.13953 (7)	0.36446 (8)	0.0195 (3)	
H59	0.0937	0.1503	0.3312	0.023*	
O1	0.23007 (7)	−0.00483 (6)	0.74263 (7)	0.0386 (3)	
C60	0.21438 (8)	−0.00046 (9)	0.66531 (10)	0.0293 (3)	
C61	0.17938 (11)	0.06610 (11)	0.61653 (13)	0.0542 (5)	
H61A	0.2148	0.0942	0.6007	0.081*	
H61B	0.1606	0.0961	0.6528	0.081*	
H61C	0.1397	0.0517	0.5640	0.081*	
C62	0.23176 (13)	−0.06106 (12)	0.61583 (13)	0.0585 (6)	
H62A	0.1895	−0.0720	0.5645	0.088*	
H62B	0.2454	−0.1046	0.6525	0.088*	
H62C	0.2718	−0.0464	0.5979	0.088*	
O2	0.69897 (6)	−0.00149 (7)	0.71163 (7)	0.0386 (3)	
C63	0.68968 (8)	0.03359 (8)	0.64648 (9)	0.0259 (3)	
C64	0.64764 (10)	0.10294 (9)	0.62856 (10)	0.0374 (4)	
H64A	0.6209	0.1073	0.6681	0.056*	
H64B	0.6137	0.1027	0.5687	0.056*	
H64C	0.6806	0.1444	0.6371	0.056*	
C65	0.71994 (9)	0.00907 (11)	0.58003 (11)	0.0425 (4)	
H65A	0.7513	−0.0332	0.6020	0.064*	
H65B	0.7478	0.0489	0.5677	0.064*	
H65C	0.6805	−0.0045	0.5269	0.064*	

### Atomic displacement parameters (Å^2^)

**Table:**

	*U*^11^	*U*^22^	*U*^33^	*U*^12^	*U*^13^	*U*^23^
N1	0.0148 (5)	0.0175 (5)	0.0178 (5)	−0.0002 (4)	0.0033 (4)	0.0029 (4)
N2	0.0163 (5)	0.0171 (5)	0.0203 (5)	0.0025 (4)	0.0060 (4)	0.0039 (4)
N3	0.0178 (5)	0.0200 (6)	0.0175 (5)	0.0054 (4)	0.0079 (4)	0.0049 (4)
N4	0.0159 (5)	0.0216 (6)	0.0144 (5)	0.0030 (4)	0.0013 (4)	0.0013 (4)
C1	0.0156 (6)	0.0169 (6)	0.0134 (5)	0.0009 (5)	0.0026 (5)	0.0004 (5)
C2	0.0220 (7)	0.0214 (7)	0.0187 (6)	0.0021 (5)	0.0039 (5)	0.0068 (5)
C3	0.0176 (6)	0.0220 (7)	0.0245 (7)	−0.0035 (5)	0.0060 (5)	0.0005 (5)
C4	0.0200 (6)	0.0210 (7)	0.0241 (7)	0.0058 (5)	0.0084 (5)	0.0033 (5)
C5	0.0166 (6)	0.0224 (7)	0.0259 (7)	0.0005 (5)	0.0060 (5)	0.0067 (6)
C6	0.0158 (6)	0.0189 (6)	0.0162 (6)	0.0030 (5)	0.0049 (5)	0.0039 (5)
C7	0.0142 (6)	0.0182 (6)	0.0176 (6)	0.0010 (5)	0.0031 (5)	0.0019 (5)
C8	0.0126 (6)	0.0219 (7)	0.0187 (6)	0.0018 (5)	0.0013 (5)	0.0034 (5)
C9	0.0273 (7)	0.0311 (8)	0.0264 (7)	0.0046 (6)	0.0047 (6)	0.0139 (6)
C10	0.0358 (8)	0.0373 (9)	0.0206 (7)	0.0198 (7)	0.0048 (6)	−0.0005 (6)
C11	0.0183 (7)	0.0327 (8)	0.0223 (7)	−0.0036 (6)	−0.0019 (5)	−0.0015 (6)
B1	0.0161 (7)	0.0149 (7)	0.0154 (6)	−0.0006 (5)	0.0057 (5)	0.0012 (5)
C12	0.0160 (6)	0.0172 (6)	0.0099 (5)	−0.0009 (5)	0.0008 (5)	0.0009 (5)
C13	0.0194 (6)	0.0204 (6)	0.0112 (5)	−0.0004 (5)	0.0027 (5)	0.0003 (5)
C14	0.0269 (7)	0.0179 (7)	0.0140 (6)	0.0008 (5)	0.0016 (5)	−0.0011 (5)
C15	0.0310 (7)	0.0181 (7)	0.0154 (6)	−0.0083 (6)	−0.0001 (5)	0.0012 (5)
C16	0.0214 (7)	0.0251 (7)	0.0167 (6)	−0.0077 (5)	0.0037 (5)	0.0015 (5)
C17	0.0172 (6)	0.0219 (7)	0.0148 (6)	−0.0009 (5)	0.0029 (5)	−0.0001 (5)
C18	0.0113 (6)	0.0184 (6)	0.0170 (6)	−0.0016 (5)	0.0049 (5)	−0.0012 (5)
C19	0.0207 (6)	0.0200 (7)	0.0187 (6)	−0.0006 (5)	0.0055 (5)	0.0016 (5)
C20	0.0217 (7)	0.0172 (7)	0.0288 (7)	−0.0005 (5)	0.0069 (6)	−0.0048 (6)
C21	0.0194 (7)	0.0288 (8)	0.0227 (7)	−0.0042 (6)	0.0098 (5)	−0.0100 (6)
C22	0.0244 (7)	0.0291 (8)	0.0168 (6)	−0.0071 (6)	0.0096 (5)	−0.0022 (5)
C23	0.0186 (6)	0.0194 (6)	0.0186 (6)	−0.0038 (5)	0.0069 (5)	−0.0008 (5)
C24	0.0186 (6)	0.0175 (6)	0.0149 (6)	−0.0026 (5)	0.0069 (5)	−0.0049 (5)
C25	0.0193 (6)	0.0229 (7)	0.0191 (6)	−0.0028 (5)	0.0053 (5)	−0.0023 (5)
C26	0.0197 (7)	0.0281 (8)	0.0250 (7)	0.0009 (6)	0.0033 (6)	−0.0053 (6)
C27	0.0189 (7)	0.0392 (9)	0.0302 (8)	−0.0065 (6)	0.0112 (6)	−0.0094 (7)
C28	0.0272 (7)	0.0311 (8)	0.0265 (7)	−0.0087 (6)	0.0145 (6)	−0.0023 (6)
C29	0.0221 (7)	0.0208 (7)	0.0214 (6)	−0.0017 (5)	0.0087 (5)	−0.0013 (5)
C30	0.0191 (6)	0.0134 (6)	0.0166 (6)	−0.0032 (5)	0.0059 (5)	0.0002 (5)
C31	0.0231 (7)	0.0188 (7)	0.0196 (6)	0.0020 (5)	0.0088 (5)	−0.0001 (5)
C32	0.0329 (8)	0.0242 (7)	0.0156 (6)	−0.0009 (6)	0.0092 (6)	0.0007 (5)
C33	0.0249 (7)	0.0255 (7)	0.0190 (6)	0.0002 (6)	0.0021 (5)	0.0066 (6)
C34	0.0186 (7)	0.0276 (8)	0.0260 (7)	0.0024 (6)	0.0076 (6)	0.0040 (6)
C35	0.0196 (6)	0.0230 (7)	0.0177 (6)	−0.0019 (5)	0.0078 (5)	0.0011 (5)
B2	0.0176 (7)	0.0137 (6)	0.0136 (6)	−0.0001 (5)	0.0061 (5)	0.0001 (5)
C36	0.0177 (6)	0.0114 (6)	0.0153 (6)	−0.0029 (5)	0.0044 (5)	−0.0006 (5)
C37	0.0183 (6)	0.0174 (6)	0.0190 (6)	−0.0030 (5)	0.0048 (5)	−0.0020 (5)
C38	0.0176 (6)	0.0172 (7)	0.0329 (7)	−0.0010 (5)	0.0030 (6)	−0.0039 (6)
C39	0.0243 (7)	0.0202 (7)	0.0270 (7)	−0.0011 (6)	−0.0046 (6)	0.0067 (6)
C40	0.0302 (8)	0.0260 (7)	0.0158 (6)	−0.0061 (6)	0.0021 (6)	0.0045 (5)
C41	0.0223 (7)	0.0182 (6)	0.0171 (6)	−0.0023 (5)	0.0060 (5)	−0.0003 (5)
C42	0.0176 (6)	0.0194 (6)	0.0110 (5)	−0.0013 (5)	0.0034 (5)	−0.0033 (5)
C43	0.0209 (6)	0.0184 (6)	0.0167 (6)	−0.0024 (5)	0.0066 (5)	−0.0039 (5)
C44	0.0231 (7)	0.0256 (7)	0.0183 (6)	−0.0079 (6)	0.0089 (5)	−0.0033 (5)
C45	0.0159 (6)	0.0360 (8)	0.0188 (6)	−0.0032 (6)	0.0063 (5)	−0.0045 (6)
C46	0.0179 (7)	0.0290 (8)	0.0239 (7)	0.0048 (6)	0.0039 (5)	0.0003 (6)
C47	0.0197 (6)	0.0253 (7)	0.0188 (6)	0.0004 (5)	0.0059 (5)	0.0025 (5)
C48	0.0136 (6)	0.0165 (6)	0.0150 (6)	−0.0025 (5)	0.0057 (5)	0.0003 (5)
C49	0.0216 (6)	0.0194 (7)	0.0186 (6)	−0.0011 (5)	0.0041 (5)	0.0023 (5)
C50	0.0233 (7)	0.0332 (8)	0.0144 (6)	−0.0061 (6)	0.0039 (5)	0.0039 (6)
C51	0.0232 (7)	0.0365 (8)	0.0162 (6)	−0.0118 (6)	0.0097 (5)	−0.0087 (6)
C52	0.0213 (7)	0.0217 (7)	0.0255 (7)	−0.0057 (5)	0.0123 (6)	−0.0086 (5)
C53	0.0153 (6)	0.0174 (6)	0.0177 (6)	−0.0022 (5)	0.0065 (5)	−0.0004 (5)
C54	0.0173 (6)	0.0152 (6)	0.0118 (5)	0.0012 (5)	0.0026 (5)	0.0011 (5)
C55	0.0244 (7)	0.0209 (7)	0.0152 (6)	−0.0032 (5)	0.0058 (5)	−0.0008 (5)
C56	0.0317 (8)	0.0277 (8)	0.0184 (6)	−0.0112 (6)	0.0081 (6)	0.0029 (6)
C57	0.0395 (9)	0.0177 (7)	0.0232 (7)	−0.0089 (6)	0.0029 (6)	0.0020 (6)
C58	0.0338 (8)	0.0166 (7)	0.0212 (7)	0.0025 (6)	0.0031 (6)	−0.0018 (5)
C59	0.0219 (7)	0.0188 (7)	0.0163 (6)	0.0026 (5)	0.0050 (5)	0.0000 (5)
O1	0.0546 (7)	0.0352 (7)	0.0290 (6)	0.0035 (6)	0.0184 (5)	0.0070 (5)
C60	0.0272 (8)	0.0311 (8)	0.0306 (8)	−0.0071 (6)	0.0117 (6)	0.0056 (6)
C61	0.0546 (12)	0.0446 (11)	0.0506 (11)	−0.0038 (9)	0.0031 (9)	0.0232 (9)
C62	0.0759 (15)	0.0629 (14)	0.0487 (11)	0.0020 (12)	0.0367 (11)	−0.0056 (10)
O2	0.0477 (7)	0.0387 (7)	0.0286 (6)	0.0024 (5)	0.0125 (5)	0.0134 (5)
C63	0.0231 (7)	0.0303 (8)	0.0204 (7)	−0.0076 (6)	0.0027 (6)	0.0012 (6)
C64	0.0495 (10)	0.0333 (9)	0.0229 (7)	0.0029 (8)	0.0047 (7)	0.0037 (6)
C65	0.0362 (9)	0.0604 (12)	0.0316 (8)	−0.0019 (8)	0.0129 (7)	−0.0047 (8)

### Geometric parameters (Å, º)

**Table:**

N1—C1	1.3331 (16)	C29—H29	0.9500
N1—C3	1.4604 (16)	C30—C35	1.3992 (18)
N1—C2	1.4649 (16)	C30—C31	1.4062 (17)
N2—C1	1.3454 (16)	C31—C32	1.3882 (19)
N2—C4	1.4601 (16)	C31—H31	0.9500
N2—C5	1.4603 (17)	C32—C33	1.384 (2)
N3—C1	1.3407 (16)	C32—H32	0.9500
N3—C6	1.4626 (16)	C33—C34	1.3882 (19)
N3—H3	0.87 (2)	C33—H33	0.9500
N4—C11	1.4930 (17)	C34—C35	1.3922 (18)
N4—C10	1.4932 (18)	C34—H34	0.9500
N4—C9	1.5033 (17)	C35—H35	0.9500
N4—C8	1.5161 (16)	B2—C54	1.6421 (19)
C2—H2A	0.9800	B2—C48	1.6456 (18)
C2—H2B	0.9800	B2—C36	1.6493 (18)
C2—H2C	0.9800	B2—C42	1.6529 (19)
C3—H3A	0.9800	C36—C41	1.4050 (17)
C3—H3B	0.9800	C36—C37	1.4061 (18)
C3—H3C	0.9800	C37—C38	1.3909 (18)
C4—H4A	0.9800	C37—H37	0.9500
C4—H4B	0.9800	C38—C39	1.388 (2)
C4—H4C	0.9800	C38—H38	0.9500
C5—H5A	0.9800	C39—C40	1.383 (2)
C5—H5B	0.9800	C39—H39	0.9500
C5—H5C	0.9800	C40—C41	1.3902 (19)
C6—C7	1.5245 (17)	C40—H40	0.9500
C6—H6A	0.9900	C41—H41	0.9500
C6—H6B	0.9900	C42—C43	1.4044 (18)
C7—C8	1.5193 (18)	C42—C47	1.4059 (18)
C7—H7A	0.9900	C43—C44	1.3948 (18)
C7—H7B	0.9900	C43—H43	0.9500
C8—H8A	0.9900	C44—C45	1.386 (2)
C8—H8B	0.9900	C44—H44	0.9500
C9—H9A	0.9800	C45—C46	1.388 (2)
C9—H9B	0.9800	C45—H45	0.9500
C9—H9C	0.9800	C46—C47	1.3954 (19)
C10—H10A	0.9800	C46—H46	0.9500
C10—H10B	0.9800	C47—H47	0.9500
C10—H10C	0.9800	C48—C53	1.4000 (18)
C11—H11A	0.9800	C48—C49	1.4034 (17)
C11—H11B	0.9800	C49—C50	1.3911 (18)
C11—H11C	0.9800	C49—H49	0.9500
B1—C18	1.6451 (18)	C50—C51	1.385 (2)
B1—C30	1.6491 (18)	C50—H50	0.9500
B1—C12	1.6496 (19)	C51—C52	1.384 (2)
B1—C24	1.6499 (18)	C51—H51	0.9500
C12—C13	1.4030 (18)	C52—C53	1.3932 (18)
C12—C17	1.4047 (18)	C52—H52	0.9500
C13—C14	1.3947 (18)	C53—H53	0.9500
C13—H13	0.9500	C54—C55	1.4025 (18)
C14—C15	1.384 (2)	C54—C59	1.4052 (18)
C14—H14	0.9500	C55—C56	1.3926 (19)
C15—C16	1.384 (2)	C55—H55	0.9500
C15—H15	0.9500	C56—C57	1.384 (2)
C16—C17	1.3953 (19)	C56—H56	0.9500
C16—H16	0.9500	C57—C58	1.384 (2)
C17—H17	0.9500	C57—H57	0.9500
C18—C23	1.4020 (17)	C58—C59	1.3914 (19)
C18—C19	1.4039 (18)	C58—H58	0.9500
C19—C20	1.3913 (19)	C59—H59	0.9500
C19—H19	0.9500	O1—C60	1.2065 (18)
C20—C21	1.384 (2)	C60—C62	1.490 (3)
C20—H20	0.9500	C60—C61	1.491 (2)
C21—C22	1.382 (2)	C61—H61A	0.9800
C21—H21	0.9500	C61—H61B	0.9800
C22—C23	1.3946 (18)	C61—H61C	0.9800
C22—H22	0.9500	C62—H62A	0.9800
C23—H23	0.9500	C62—H62B	0.9800
C24—C29	1.4005 (18)	C62—H62C	0.9800
C24—C25	1.4058 (18)	O2—C63	1.2109 (17)
C25—C26	1.3923 (19)	C63—C64	1.491 (2)
C25—H25	0.9500	C63—C65	1.496 (2)
C26—C27	1.385 (2)	C64—H64A	0.9800
C26—H26	0.9500	C64—H64B	0.9800
C27—C28	1.386 (2)	C64—H64C	0.9800
C27—H27	0.9500	C65—H65A	0.9800
C28—C29	1.3950 (19)	C65—H65B	0.9800
C28—H28	0.9500	C65—H65C	0.9800
			
C1—N1—C3	121.42 (11)	C27—C28—C29	120.09 (14)
C1—N1—C2	122.51 (11)	C27—C28—H28	120.0
C3—N1—C2	115.15 (10)	C29—C28—H28	120.0
C1—N2—C4	122.78 (11)	C28—C29—C24	122.98 (13)
C1—N2—C5	122.18 (11)	C28—C29—H29	118.5
C4—N2—C5	114.95 (10)	C24—C29—H29	118.5
C1—N3—C6	126.20 (11)	C35—C30—C31	114.92 (11)
C1—N3—H3	115.5 (11)	C35—C30—B1	122.90 (11)
C6—N3—H3	118.2 (11)	C31—C30—B1	122.00 (11)
C11—N4—C10	109.15 (11)	C32—C31—C30	123.11 (13)
C11—N4—C9	108.42 (10)	C32—C31—H31	118.4
C10—N4—C9	108.75 (12)	C30—C31—H31	118.4
C11—N4—C8	110.98 (11)	C33—C32—C31	120.08 (12)
C10—N4—C8	111.47 (10)	C33—C32—H32	120.0
C9—N4—C8	107.99 (10)	C31—C32—H32	120.0
N1—C1—N3	118.96 (11)	C32—C33—C34	118.83 (12)
N1—C1—N2	120.53 (11)	C32—C33—H33	120.6
N3—C1—N2	120.51 (12)	C34—C33—H33	120.6
N1—C2—H2A	109.5	C33—C34—C35	120.22 (13)
N1—C2—H2B	109.5	C33—C34—H34	119.9
H2A—C2—H2B	109.5	C35—C34—H34	119.9
N1—C2—H2C	109.5	C34—C35—C30	122.85 (12)
H2A—C2—H2C	109.5	C34—C35—H35	118.6
H2B—C2—H2C	109.5	C30—C35—H35	118.6
N1—C3—H3A	109.5	C54—B2—C48	111.18 (10)
N1—C3—H3B	109.5	C54—B2—C36	104.60 (10)
H3A—C3—H3B	109.5	C48—B2—C36	112.69 (10)
N1—C3—H3C	109.5	C54—B2—C42	111.13 (10)
H3A—C3—H3C	109.5	C48—B2—C42	105.75 (10)
H3B—C3—H3C	109.5	C36—B2—C42	111.63 (10)
N2—C4—H4A	109.5	C41—C36—C37	114.83 (11)
N2—C4—H4B	109.5	C41—C36—B2	122.53 (11)
H4A—C4—H4B	109.5	C37—C36—B2	122.35 (11)
N2—C4—H4C	109.5	C38—C37—C36	123.12 (12)
H4A—C4—H4C	109.5	C38—C37—H37	118.4
H4B—C4—H4C	109.5	C36—C37—H37	118.4
N2—C5—H5A	109.5	C39—C38—C37	119.94 (13)
N2—C5—H5B	109.5	C39—C38—H38	120.0
H5A—C5—H5B	109.5	C37—C38—H38	120.0
N2—C5—H5C	109.5	C40—C39—C38	118.82 (12)
H5A—C5—H5C	109.5	C40—C39—H39	120.6
H5B—C5—H5C	109.5	C38—C39—H39	120.6
N3—C6—C7	109.86 (10)	C39—C40—C41	120.55 (13)
N3—C6—H6A	109.7	C39—C40—H40	119.7
C7—C6—H6A	109.7	C41—C40—H40	119.7
N3—C6—H6B	109.7	C40—C41—C36	122.70 (13)
C7—C6—H6B	109.7	C40—C41—H41	118.7
H6A—C6—H6B	108.2	C36—C41—H41	118.7
C8—C7—C6	108.70 (10)	C43—C42—C47	114.90 (12)
C8—C7—H7A	109.9	C43—C42—B2	122.91 (11)
C6—C7—H7A	109.9	C47—C42—B2	122.16 (11)
C8—C7—H7B	109.9	C44—C43—C42	122.91 (13)
C6—C7—H7B	110.0	C44—C43—H43	118.5
H7A—C7—H7B	108.3	C42—C43—H43	118.5
N4—C8—C7	115.19 (10)	C45—C44—C43	120.34 (13)
N4—C8—H8A	108.5	C45—C44—H44	119.8
C7—C8—H8A	108.5	C43—C44—H44	119.8
N4—C8—H8B	108.5	C44—C45—C46	118.58 (12)
C7—C8—H8B	108.5	C44—C45—H45	120.7
H8A—C8—H8B	107.5	C46—C45—H45	120.7
N4—C9—H9A	109.5	C45—C46—C47	120.39 (13)
N4—C9—H9B	109.5	C45—C46—H46	119.8
H9A—C9—H9B	109.5	C47—C46—H46	119.8
N4—C9—H9C	109.5	C46—C47—C42	122.74 (13)
H9A—C9—H9C	109.5	C46—C47—H47	118.6
H9B—C9—H9C	109.5	C42—C47—H47	118.6
N4—C10—H10A	109.5	C53—C48—C49	114.85 (11)
N4—C10—H10B	109.5	C53—C48—B2	123.16 (11)
H10A—C10—H10B	109.5	C49—C48—B2	121.75 (11)
N4—C10—H10C	109.5	C50—C49—C48	122.89 (13)
H10A—C10—H10C	109.5	C50—C49—H49	118.6
H10B—C10—H10C	109.5	C48—C49—H49	118.6
N4—C11—H11A	109.5	C51—C50—C49	120.29 (13)
N4—C11—H11B	109.5	C51—C50—H50	119.9
H11A—C11—H11B	109.5	C49—C50—H50	119.9
N4—C11—H11C	109.5	C52—C51—C50	118.75 (12)
H11A—C11—H11C	109.5	C52—C51—H51	120.6
H11B—C11—H11C	109.5	C50—C51—H51	120.6
C18—B1—C30	111.11 (10)	C51—C52—C53	120.12 (13)
C18—B1—C12	113.08 (10)	C51—C52—H52	119.9
C30—B1—C12	105.44 (10)	C53—C52—H52	119.9
C18—B1—C24	104.53 (10)	C52—C53—C48	123.08 (12)
C30—B1—C24	112.57 (10)	C52—C53—H53	118.5
C12—B1—C24	110.30 (10)	C48—C53—H53	118.5
C13—C12—C17	115.10 (12)	C55—C54—C59	115.10 (12)
C13—C12—B1	123.28 (11)	C55—C54—B2	122.45 (11)
C17—C12—B1	121.56 (11)	C59—C54—B2	122.25 (11)
C14—C13—C12	122.80 (12)	C56—C55—C54	122.69 (13)
C14—C13—H13	118.6	C56—C55—H55	118.7
C12—C13—H13	118.6	C54—C55—H55	118.7
C15—C14—C13	120.18 (13)	C57—C56—C55	120.27 (13)
C15—C14—H14	119.9	C57—C56—H56	119.9
C13—C14—H14	119.9	C55—C56—H56	119.9
C14—C15—C16	118.98 (13)	C56—C57—C58	118.98 (13)
C14—C15—H15	120.5	C56—C57—H57	120.5
C16—C15—H15	120.5	C58—C57—H57	120.5
C15—C16—C17	120.19 (13)	C57—C58—C59	120.10 (13)
C15—C16—H16	119.9	C57—C58—H58	120.0
C17—C16—H16	119.9	C59—C58—H58	120.0
C16—C17—C12	122.71 (13)	C58—C59—C54	122.84 (13)
C16—C17—H17	118.6	C58—C59—H59	118.6
C12—C17—H17	118.6	C54—C59—H59	118.6
C23—C18—C19	114.69 (12)	O1—C60—C62	120.61 (16)
C23—C18—B1	125.51 (11)	O1—C60—C61	121.55 (16)
C19—C18—B1	119.45 (11)	C62—C60—C61	117.81 (16)
C20—C19—C18	123.52 (12)	C60—C61—H61A	109.5
C20—C19—H19	118.2	C60—C61—H61B	109.5
C18—C19—H19	118.2	H61A—C61—H61B	109.5
C21—C20—C19	119.85 (13)	C60—C61—H61C	109.5
C21—C20—H20	120.1	H61A—C61—H61C	109.5
C19—C20—H20	120.1	H61B—C61—H61C	109.5
C22—C21—C20	118.63 (12)	C60—C62—H62A	109.5
C22—C21—H21	120.7	C60—C62—H62B	109.5
C20—C21—H21	120.7	H62A—C62—H62B	109.5
C21—C22—C23	120.87 (13)	C60—C62—H62C	109.5
C21—C22—H22	119.6	H62A—C62—H62C	109.5
C23—C22—H22	119.6	H62B—C62—H62C	109.5
C22—C23—C18	122.43 (13)	O2—C63—C64	121.79 (14)
C22—C23—H23	118.8	O2—C63—C65	121.31 (15)
C18—C23—H23	118.8	C64—C63—C65	116.89 (14)
C29—C24—C25	114.95 (12)	C63—C64—H64A	109.5
C29—C24—B1	124.08 (11)	C63—C64—H64B	109.5
C25—C24—B1	120.85 (11)	H64A—C64—H64B	109.5
C26—C25—C24	122.87 (13)	C63—C64—H64C	109.5
C26—C25—H25	118.6	H64A—C64—H64C	109.5
C24—C25—H25	118.6	H64B—C64—H64C	109.5
C27—C26—C25	120.22 (13)	C63—C65—H65A	109.5
C27—C26—H26	119.9	C63—C65—H65B	109.5
C25—C26—H26	119.9	H65A—C65—H65B	109.5
C26—C27—C28	118.85 (13)	C63—C65—H65C	109.5
C26—C27—H27	120.6	H65A—C65—H65C	109.5
C28—C27—H27	120.6	H65B—C65—H65C	109.5
			
C3—N1—C1—N3	−22.25 (18)	C35—C30—C31—C32	−0.01 (19)
C2—N1—C1—N3	146.25 (12)	B1—C30—C31—C32	175.37 (12)
C3—N1—C1—N2	157.48 (12)	C30—C31—C32—C33	−0.2 (2)
C2—N1—C1—N2	−34.03 (18)	C31—C32—C33—C34	0.4 (2)
C6—N3—C1—N1	151.54 (12)	C32—C33—C34—C35	−0.5 (2)
C6—N3—C1—N2	−28.19 (19)	C33—C34—C35—C30	0.3 (2)
C4—N2—C1—N1	−29.95 (18)	C31—C30—C35—C34	−0.07 (19)
C5—N2—C1—N1	146.47 (12)	B1—C30—C35—C34	−175.41 (12)
C4—N2—C1—N3	149.77 (12)	C54—B2—C36—C41	−90.16 (14)
C5—N2—C1—N3	−33.80 (18)	C48—B2—C36—C41	148.93 (12)
C1—N3—C6—C7	−151.03 (12)	C42—B2—C36—C41	30.10 (16)
N3—C6—C7—C8	−172.13 (11)	C54—B2—C36—C37	83.36 (14)
C11—N4—C8—C7	61.70 (14)	C48—B2—C36—C37	−37.55 (16)
C10—N4—C8—C7	−60.19 (15)	C42—B2—C36—C37	−156.38 (11)
C9—N4—C8—C7	−179.59 (11)	C41—C36—C37—C38	−1.87 (18)
C6—C7—C8—N4	177.55 (11)	B2—C36—C37—C38	−175.85 (12)
C18—B1—C12—C13	−140.56 (11)	C36—C37—C38—C39	0.4 (2)
C30—B1—C12—C13	97.85 (13)	C37—C38—C39—C40	1.0 (2)
C24—B1—C12—C13	−23.93 (16)	C38—C39—C40—C41	−0.8 (2)
C18—B1—C12—C17	42.46 (15)	C39—C40—C41—C36	−0.8 (2)
C30—B1—C12—C17	−79.12 (13)	C37—C36—C41—C40	2.09 (19)
C24—B1—C12—C17	159.10 (11)	B2—C36—C41—C40	176.06 (12)
C17—C12—C13—C14	−1.82 (17)	C54—B2—C42—C43	148.78 (11)
B1—C12—C13—C14	−178.97 (11)	C48—B2—C42—C43	−90.46 (13)
C12—C13—C14—C15	0.39 (19)	C36—B2—C42—C43	32.43 (16)
C13—C14—C15—C16	1.18 (19)	C54—B2—C42—C47	−33.21 (15)
C14—C15—C16—C17	−1.20 (19)	C48—B2—C42—C47	87.55 (14)
C15—C16—C17—C12	−0.35 (19)	C36—B2—C42—C47	−149.56 (11)
C13—C12—C17—C16	1.80 (17)	C47—C42—C43—C44	3.05 (18)
B1—C12—C17—C16	179.00 (11)	B2—C42—C43—C44	−178.81 (11)
C30—B1—C18—C23	140.83 (12)	C42—C43—C44—C45	0.00 (19)
C12—B1—C18—C23	22.49 (17)	C43—C44—C45—C46	−2.59 (19)
C24—B1—C18—C23	−97.51 (13)	C44—C45—C46—C47	2.0 (2)
C30—B1—C18—C19	−46.28 (15)	C45—C46—C47—C42	1.3 (2)
C12—B1—C18—C19	−164.62 (11)	C43—C42—C47—C46	−3.69 (18)
C24—B1—C18—C19	75.38 (14)	B2—C42—C47—C46	178.15 (12)
C23—C18—C19—C20	0.58 (19)	C54—B2—C48—C53	−142.90 (12)
B1—C18—C19—C20	−173.05 (12)	C36—B2—C48—C53	−25.84 (16)
C18—C19—C20—C21	−0.6 (2)	C42—B2—C48—C53	96.38 (13)
C19—C20—C21—C22	0.2 (2)	C54—B2—C48—C49	43.04 (16)
C20—C21—C22—C23	0.2 (2)	C36—B2—C48—C49	160.10 (11)
C21—C22—C23—C18	−0.2 (2)	C42—B2—C48—C49	−77.69 (14)
C19—C18—C23—C22	−0.21 (18)	C53—C48—C49—C50	−0.69 (19)
B1—C18—C23—C22	172.97 (12)	B2—C48—C49—C50	173.84 (12)
C18—B1—C24—C29	−96.72 (13)	C48—C49—C50—C51	−0.7 (2)
C30—B1—C24—C29	23.98 (17)	C49—C50—C51—C52	1.1 (2)
C12—B1—C24—C29	141.44 (12)	C50—C51—C52—C53	−0.2 (2)
C18—B1—C24—C25	79.09 (14)	C51—C52—C53—C48	−1.3 (2)
C30—B1—C24—C25	−160.21 (11)	C49—C48—C53—C52	1.68 (18)
C12—B1—C24—C25	−42.75 (15)	B2—C48—C53—C52	−172.76 (12)
C29—C24—C25—C26	−1.84 (19)	C48—B2—C54—C55	34.37 (16)
B1—C24—C25—C26	−178.01 (12)	C36—B2—C54—C55	−87.52 (13)
C24—C25—C26—C27	0.3 (2)	C42—B2—C54—C55	151.87 (11)
C25—C26—C27—C28	1.2 (2)	C48—B2—C54—C59	−150.98 (11)
C26—C27—C28—C29	−1.1 (2)	C36—B2—C54—C59	87.12 (13)
C27—C28—C29—C24	−0.5 (2)	C42—B2—C54—C59	−33.48 (16)
C25—C24—C29—C28	1.95 (19)	C59—C54—C55—C56	0.49 (18)
B1—C24—C29—C28	177.98 (12)	B2—C54—C55—C56	175.49 (12)
C18—B1—C30—C35	−27.89 (16)	C54—C55—C56—C57	0.6 (2)
C12—B1—C30—C35	94.97 (14)	C55—C56—C57—C58	−0.9 (2)
C24—B1—C30—C35	−144.73 (12)	C56—C57—C58—C59	0.1 (2)
C18—B1—C30—C31	157.09 (11)	C57—C58—C59—C54	1.1 (2)
C12—B1—C30—C31	−80.05 (14)	C55—C54—C59—C58	−1.32 (18)
C24—B1—C30—C31	40.25 (16)	B2—C54—C59—C58	−176.33 (12)

### Hydrogen-bond geometry (Å, º)

Cg1, Cg2, Cg3, Cg4 and Cg5 are the centroids of the C42–C47, C48–C53, C18–C23, C36–C41 and C54–C59 rings, respectively.

**Table:**

*D*—H···*A*	*D*—H	H···*A*	*D*···*A*	*D*—H···*A*
N3—H3···O1^i^	0.87 (2)	2.18 (2)	2.914 (2)	142 (2)
C11—H11*A*···O2^ii^	0.98	2.48	3.368 (2)	151
C10—H10*C*···*Cg*1	0.98	2.82	3.693 (1)	150
C8—H8*B*···*Cg*2	0.99	2.82	3.510 (2)	127
C3—H3*A*···*Cg*3^iii^	0.98	2.79	3.359 (1)	118
C2—H2*C*···*Cg*4^iv^	0.98	2.61	3.453 (1)	144
C2—H2*A*···*Cg*5^iv^	0.98	2.59	3.393 (1)	140

Symmetry codes: (i) *x*, −*y*+1/2, *z*−1/2; (ii) −*x*+1, *y*+1/2, −*z*+3/2; (iii) −*x*+1, −*y*+1, −*z*+1; (iv) −*x*, *y*+1/2, −*z*+1/2.
